# A retrospective study on triggering factors and management of epistaxis – our experience in a tertiary ENT clinic

**DOI:** 10.25122/jml-2025-0107

**Published:** 2025-08

**Authors:** Raluca Oana Pulpă, Ruxandra Oana Aliuș, Andreea Rusescu, Irina-Gabriela Ioniță, Răzvan Hainăroșie, Cătălina Voiosu, Viorel Zainea

**Affiliations:** 1 ENT Department, Carol Davila University of Medicine and Pharmacy, Bucharest, Romania; 2 ENT Department, Prof. Dr. D. Hociota Institute of Phonoaudiology and Functional ENT Surgery, Bucharest, Romania

**Keywords:** epistaxis, ENT emergency, triggering factors, bleeding, management

## Abstract

Epistaxis is one of the most frequent ENT emergencies, with complex etiologies ranging from local trauma to systemic conditions. This retrospective study analyzed 1,173 patients who presented with epistaxis at a tertiary ENT center over 5 years. Of these, 260 required admissions. The most common triggering factors were hypertension, anticoagulant/antiaggregant therapy, and postoperative complications. A significant portion (38.46%) of cases were idiopathic. Recurrent bleeding was often associated with high blood pressure and male gender, particularly in the 61–70-year age group. Hereditary hemorrhagic telangiectasia was diagnosed in 11 patients, all requiring repeated interventions. Most anterior nasal bleedings had a good response to conservative measures. Posterior or severe cases of epistaxis may require surgical interventions such as cauterization or argon plasma coagulation. The purpose of this paper is to highlight the importance of individualized management, taking into account the location, etiology, and severity. Proper control of cardiovascular comorbidities and careful postoperative monitoring are essential to reducing recurrence and complications associated with epistaxis.

## INTRODUCTION

Epistaxis is among the most common symptoms encountered in the emergency department. Epidemiological studies suggest that approximately 60% of the population will experience at least one episode of nosebleed during their lifetime [1-3]. However, only about 10% of cases require medical attention, as most episodes are self-limiting [4].

Nearly 90% of patients present with anterior epistaxis originating from Kiesselbach’s plexus on the nasal septum [5]. The anatomical characteristics of this region, namely, the vascular anastomosis between branches of the internal and external carotid systems, coupled with the limited contractile fibers and sparse perivascular connective tissue of the nasal mucosal vessels, predispose it to vessel rupture and bleeding. Posterior epistaxis, which accounts for approximately 10% of cases, is typically arterial in origin and often poses greater challenges in achieving hemostasis [4,5].

Multiple factors may trigger epistaxis, including local, systemic, meteorological, and iatrogenic causes. In the majority of cases, approximately 85%, a specific etiology cannot be identified, and these are classified as idiopathic or spontaneous episodes. Secondary epistaxis, in contrast, refers to cases with a clear precipitating factor such as trauma, recent nasal surgery, or coagulation abnormalities. Local causes include trauma, recent nasal surgery, self-inflicted wounds (digital trauma), inhalation of irritating agents, atmospheric pressure variations, acute rhinosinusal disease, septal perforation, malignant or benign tumors, and foreign bodies in the nasal cavity [6]. The category of systemic causes is more comprehensive, including hypertension, age, cardiopulmonary and vascular disease, bleeding diathesis, nonsteroidal anti-inflammatory drugs, and anticoagulants. Hypertension alone is not considered a direct precipitant; rather, underlying vasculopathy combined with anticoagulant therapy increases the risk of nasal bleeding [7]. There also seems to be a correlation between the time of day and the occurrence of nosebleeds, with episodes being more frequent in the morning and late evening [8]. Seasonality plays a role as well, with anterior epistaxis more commonly reported during the winter months, largely due to mucosal dryness caused by indoor heating and concurrent upper respiratory tract inflammation [5,9].

Given that nasal bleeding is among the most common emergencies in otorhinolaryngology, we conducted a cross-sectional study to investigate the conditions that favor or trigger epistaxis, as well as the therapeutic techniques and technologies used in its management and their effectiveness. The objective of this article is to identify the leading causes of epistaxis and to evaluate the treatment strategies applied in patients who required medical care in our hospital over 5 years.

## MATERIAL AND METHODS

This cross-sectional, retrospective study analyzed patients who presented with epistaxis at the Prof. Dr. D. Hociotă Institute of Phonoaudiology and ENT Surgery, Bucharest, over 5 years (January 2018–December 2022. The study population included patients admitted with a primary diagnosis of epistaxis or who developed epistaxis during hospitalization. Exclusion criteria were patients treated only in the emergency room without admission, pediatric cases, and incomplete medical records. Out of 1,173 patients diagnosed with epistaxis, 260 hospitalized patients met the inclusion criteria and were analyzed in detail.

Data collected included demographic and social factors, age, gender, blood pressure, comorbidities, chronic medication, bleeding site, identified causes of epistaxis onset, recurrence, and medical or surgical interventions used to achieve hemostasis. Information was obtained from the hospital’s electronic database and patient observation sheets. All patients were informed about the future use of their data for medical research, scientific, and educational purposes, and signed an informed consent form.

Patients were stratified by age, gender, comorbidities, and anticoagulant use. Comparative analyses were performed using the Pearson correlation coefficient, with statistical significance defined as *P* < 0.05. The main goal of the study was to analyze the most frequently encountered triggering factors for epistaxis, the interaction between patients' comorbidities and chronic treatment, and how this influences the precipitation of nasal bleedings.

### Data analysis

Data were analyzed using IBM SPSS Statistics 25 and illustrated with Microsoft Office Excel/Word 2021. Quantitative variables were tested for normality with the Shapiro–Wilk test and expressed as mean ± standard deviation (SD) or median with interquartile range (IQR). Non-normally distributed quantitative variables were compared between groups using the Mann–Whitney U test. Qualitative variables were expressed as counts and percentages, and comparisons between groups were performed using Fisher’s Exact Test. Z-tests with Bonferroni correction were applied for detailed analyses of contingency tables. A two-tailed *P* value < 0.05 was considered statistically significant.

## RESULTS

From a total of 1173 patients who presented with epistaxis during the study period, only 260 (22.2%) required hospitalization. This confirms that most cases can be managed in the emergency department, while only a minority need extensive medical or surgical intervention, a proportion consistent with international literature [10].

Table 1 summarizes the characteristics of the study population. The mean age was 54.95 ± 18.06 years (median 55.5), with nearly half of patients (48.8%) aged 35–65 years, and 34.6% over 65 years. Men represented 60% of cases. The mean hospitalization period was 6.55 ± 3.15 days (median 6), and 41.5% of patients were hospitalized ≥6 days. Mean hemoglobin concentration was 11.58 ± 2.7 g/dL. Hypertension (63.8%) and cardiovascular disease (43.5%) were the most frequent comorbidities; 11.9% had diabetes mellitus, and 46.9% were on anticoagulant therapy. Recurrent epistaxis occurred in 71.9% of patients, bilateral bleeding in 22.3%, and postoperative bleeding in 15.4%. Fourteen percent had a history of oncologic disease. Anterior epistaxis was the most common presentation (49.6%), while 44.6% of all cases were classified as severe. The main interventions were anterior nasal packing (49.6%) and electrocautery (28.8%).

**Table 1:** Patient characteristics

Parameter	Value
**Age (Mean ± SD, Median (IQR))**	54.95 ± 18.06, 55.5 (42-68.75)
**Age group (*n*, %)**	
< 35 years	43 (16.5%)
35-65 years	127 (48.8%)
> 65 years	90 (34.6%)
**Gender (*n*, %)**	104 (40%) Female, 156 (60%) Male
**Hospitalization period (Mean ± SD, Median (IQR))**	6.55 ± 3.15, 6 (4.75-8)
**Hemoglobin (Mean ± SD, Median (IQR))**	11.58 ± 2.7, 11.8 (9.5-13.7)
**Hospitalization period ≥ 7 days (*n*, %)**	107 (41.5%)
**Hypertension (*n*, %)**	166 (63.8%)
**Cardiovascular conditions history (*n*, %)**	113 (43.5%)
**Diabetes mellitus (*n*, %)**	31 (11.9%)
**Anticoagulant therapy (*n*, %)**	122 (46.9%)
**Recurrent epistaxis (*n*, %)**	187 (71.9%)
**Bilateral epistaxis (*n*, %)**	57 (22.3%)
**Postoperative epistaxis (*n*, %)**	40 (15.4%)
**Oncologic conditions history (*n*, %)**	37 (14.2%)
**Location of epistaxis (*n*, %)**	
Anterior	129 (49.6%)
Antero-posterior	41 (15.8%)
Diffuse	20 (7.7%)
Posterior	48 (18.5%)
Nasal cavity tumor	22 (8.5%)
Severe epistaxis (*n*, %)	116 (44.6%)
**Type of intervention (*n*, %)**	
None – Spontaneous stop of bleeding	20 (7.7%)
Anterior nasal packing	129 (49.6%)
Administration of vasoconstrictor drugs	31 (11.9%)
Chemical cautery	5 (1.9%)
Electrocautery	75 (28.8%)

**Figure 1:**
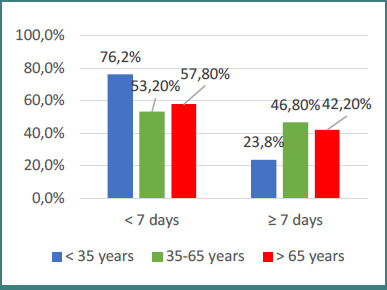
Distribution of patients by age group and hospitalization duration

**Figure 2:**
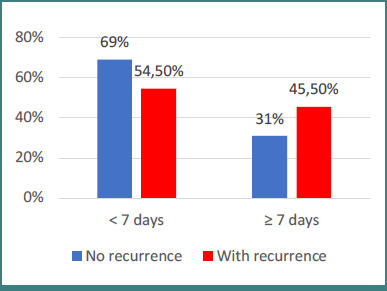
Distribution of patients by epistaxis recurrence status and hospitalization duration

Most of the analyzed characteristics were not significantly different between patients grouped by hospitalization period, except for age and recurrence (Figures 1 and 2). For age (*P* = 0.028), Z-tests with Bonferroni correction showed that patients with a hospitalization period of seven days or more were significantly more often in the 35–65 years group and less frequently under 35 years (46.8% vs 23.8%). For recurrence (*P* = 0.047), patients with a hospitalization period of seven days or more were significantly more often associated with epistaxis recurrence (45.5% vs 31%).

When analyzing the distribution of patients according to hospitalization period and epistaxis location, intervention, severity, and hemoglobin level, several significant differences were observed. Epistaxis locations were significantly different between groups (*P* = 0.015). Z-tests with Bonferroni correction showed that anterior epistaxis was significantly more frequent in patients hospitalized for less than seven days (58.3% vs 37.4%). Interventions also differed significantly between groups (*P* = 0.020), with vasoconstrictor administration more frequent in patients with shorter hospitalization (15.9% vs 6.5%) and electrocautery more frequent in those with more extended hospitalization (38.3% vs 21.9%).

Patients with severe epistaxis were significantly more often hospitalized for 7 days or more compared with those with non-severe bleeding (58.9% vs 34.4%, *P* < 0.001). Hemoglobin values also differed significantly: patients hospitalized for seven days or more had lower levels (median = 10.4 g/dL, IQR 8.9–13.2) compared with patients with shorter hospitalization (median = 12.5 g/dL, IQR 10.5–13.9; *p* = 0.001).

**Figure 3:**
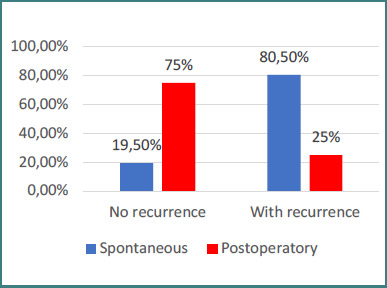
Distribution of patients by type of epistaxis and recurrence

**Figure 4:**
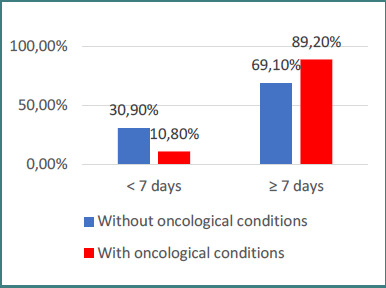
Distribution of patients by oncological history and recurrence status

Data from Figures 3 and 4 illustrate the distribution of patients according to recurrence and key characteristics. Patients with recurrent epistaxis were significantly less often associated with postoperative bleeding and more frequently with spontaneous epistaxis (80.5% vs 25%, *p* < 0.001). In addition, recurrence was significantly more common among patients with oncological conditions compared to those without (89.2% vs 69.1%, *P* = 0.010).

**Figure 5:**
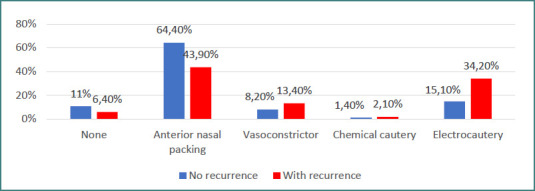
Distribution of patients by intervention type and recurrence status

Data from Figure 5 illustrates the distribution of patients according to recurrence status, epistaxis location, intervention, severity, and hemoglobin level, highlighting several significant associations.

Epistaxis location differed significantly between groups (*P* = 0.002). Z-tests with Bonferroni correction showed that posterior epistaxis was less frequently associated with recurrence (14.4% vs 28.8%), whereas nasal cavity tumors were more frequently associated with recurrence (11.2% vs 1.4%). Epistaxis interventions differed significantly between groups (*P* = 0.005). Z-tests with Bonferroni correction showed that anterior nasal packing was less frequently associated with recurrence (43.9% vs 64.4%), whereas electrocautery was more frequently associated with recurrence (34.2% vs 15.1%).

Analyzing the distribution of patients according to severity, epistaxis location, and intervention, several significant associations were observed. Epistaxis locations were significantly different between groups (*P* < 0.001). Z-tests with Bonferroni correction showed that patients with anterior epistaxis were significantly more likely to have non-severe epistaxis (66% vs. 29.3%), while patients with posterior epistaxis were significantly more likely to have severe epistaxis (32.8% vs. 6.9%). Interventions also varied significantly between groups (*P* < 0.001). Patients with no intervention (13.9% vs 0%), vasoconstrictor administration (21.5% vs 0%), or chemical cautery (3.5% vs 0%) were more frequently associated with non-severe epistaxis. By contrast, electrocautery was significantly more common in severe cases (54.3% vs 8.3%).

### Treatment options

Epistaxis was managed using a stepwise treatment protocol. Initial measures consisted of nasal alar compression and the topical application of vasoconstrictors. If bleeding persisted, anterior nasal packing with a Merocele nasal dressing was performed. For clearly localized anterior bleeding sites, chemical cauterization with silver nitrate was applied. Posterior or refractory bleeds were managed endoscopically using bipolar or monopolar electrocautery. In selected cases, particularly patients with hereditary hemorrhagic telangiectasia, argon plasma coagulation was employed. Treatment decisions were individualized according to bleeding severity, location, and patient comorbidities.

**Figure 6:**
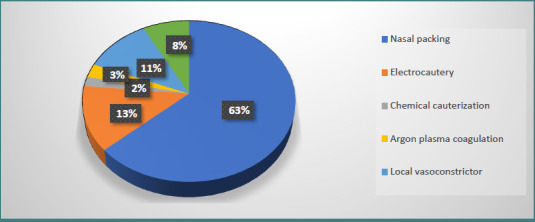
Treatment options in epistaxis management

Nasal packing remains the most frequently used treatment for anterior or diffuse epistaxis (Figure 6). It is highly effective, inexpensive, and straightforward to perform in the ENT emergency setting. Depending on the severity of bleeding and patient comorbidities, nasal packing may be maintained for 48–72 hours, or longer if required.

Silver nitrate cauterization using silver nitrate applicators (75% silver nitrate and 25% potassium nitrate) is commonly used for clearly identified anterior bleeding sources. If the vessel is small enough, the method is effective. Still, it should be used cautiously on small areas to prevent septal perforation, as the chemical reaction persists even after removing the nitrate agent.

We prefer bipolar over monopolar cauterization for posterior epistaxis, most often of arterial origin, or cases unresponsive to nasal packing. Bipolar cauterization is a reliable treatment option with a lower risk of septal perforation.

For patients with hereditary hemorrhagic telangiectasia, argon plasma coagulation is the preferred treatment. It offers reasonable control of nasal bleeding with no significant complications. Its thermal effect extends only 1–2 mm in depth, thereby protecting the underlying nasal mucosa.

## DISCUSSION

Epistaxis remains one of the most frequent emergencies encountered in otolaryngology, with a wide spectrum of etiological factors and therapeutic approaches. Although the majority of nosebleeds are self-limited and can be managed conservatively, a significant proportion of cases require specialized medical or surgical intervention, particularly in the presence of comorbidities or persistent bleeding. Despite its common occurrence, the underlying mechanisms and optimal management strategies for epistaxis continue to be a subject of clinical interest, particularly in relation to systemic factors such as hypertension, anticoagulant therapy, and postoperative status.

The present study offers a retrospective analysis of 1,173 patients presenting with epistaxis over five years in a tertiary ENT center, focusing on the identification of major triggering factors, the correlation with patient comorbidities, and the spectrum of therapeutic interventions employed. Our findings revealed that hypertension and cardiovascular-related treatments, along with postoperative complications, were among the most frequently associated factors with clinically significant epistaxis requiring hospitalization. Additionally, a notable percentage of cases remained idiopathic, underlining the complexity and multifactorial nature of nasal bleeding.

This discussion aims to contextualize these findings within the broader body of literature, to explore the implications of comorbid conditions and pharmacological treatments in the pathophysiology of epistaxis, and to evaluate the efficacy of current treatment modalities. Furthermore, we aim to highlight clinical considerations for individualized management strategies, especially in high-risk patient populations such as those receiving anticoagulants or those with hereditary hemorrhagic telangiectasia. The following sections will systematically examine each major finding, compare our data to existing evidence, and consider possible directions for clinical practice and future research.

### Hypertension

Multiple studies have investigated the association between nasal bleeding and hypertension, but the relationship remains unclear. Epistaxis does not appear to be a direct consequence of elevated blood pressure; rather, the underlying vascular changes and the cardiovascular therapies used for these conditions may contribute to its occurrence [11].

In our analysis, we found significant correlations between hypertension, cardiovascular comorbidities, and epistaxis. Patients with cardiovascular conditions were significantly more frequently associated with severe epistaxis. Arterial hypertension induces structural changes in both microvessels and larger-caliber vessels as an adaptive response to elevated blood pressure. A postmortem study revealed degenerative fibrous changes in the nasal vessels of patients with hypertension [12]. Over time, these adaptive mechanisms are overwhelmed, leading to vessel wall weakening and rupture [13-16].

Atherosclerotic changes, including plaque deposition in medium and large arteries and thickening of arterioles and small arteries, further predispose to vascular rupture throughout the body [17,18]. Arterial high blood pressure induces oxidative stress that damages the vascular endothelium [19]. The most frequent site of bleeding in hypertensive patients remains Kiesselbach’s area. Proper and constant management of hypertension is essential to prevent repetitive epistaxis from the anterior part of the nasal septum.

On the other hand, patients with a history of high blood pressure and severe epistaxis have a higher risk of atherosclerotic cardiovascular disease [20]. In this regard, otolaryngologists are essential in diagnosing and referring the patient to cardiologists for proper antihypertensive and antiaggregant therapy. It should also be noted that before the age of 50, women benefit from vascular protection provided by progesterone. After menopause, when progesterone levels decline, women’s risk of developing cardiovascular disease increases to a level comparable with that of men.

### Anticoagulant and antiplatelet drugs

More than half of patients with epistaxis have underlying cardiovascular disease and are receiving antithrombotic therapy, including antiplatelet agents, vitamin K antagonists, direct oral anticoagulants, or combinations thereof. Although vascular fragility associated with hypertension and impaired hemostasis due to anticoagulation can complicate bleeding control, anticoagulant therapy should not be discontinued unless the epistaxis is massive or refractory to treatment. In cases of overdose or uncontrollable bleeding, dose adjustment should be considered in consultation with a cardiologist or hematologist [21].

Hemostasis typically recovers within 10 days after cessation of antiplatelet therapy; thus, stopping these agents in the context of acute epistaxis is generally ineffective. For patients with uncontrollable epistaxis while on antiplatelet treatment, therapy discontinuation combined with platelet transfusion is a reasonable approach [22]. When evaluating the suspension of anticoagulant therapy, the thrombotic risk in cardiovascular patients must also be considered. Vitamin K antagonists should be withheld, and administration of a reversal agent considered, only in cases where epistaxis cannot be controlled by standard measures.

### Postoperatively epistaxis

Postoperative epistaxis is uncommon. However, ENT surgeons may occasionally encounter significant hemorrhage requiring prompt intervention. The surgical procedures most commonly associated with postoperative nasal bleeding include rhinosinus surgery and endoscopic endonasal transsphenoidal approaches.

In most cases, the bleeding is arterial, often diffuse, and sometimes difficult to localize. Anterior nasal packing is generally ineffective in these situations [23,24], since the usual source lies in the posterior nasal cavity, most commonly the sphenopalatine artery or its branches. Posterior nasal packing can sometimes provide temporary control, but if bleeding persists, the patient should be taken for endoscopic exploration under general anesthesia. This allows identification of the source and application of definitive surgical treatment [25].

### Trauma

In pediatric patients, trauma produced by digital manipulation or the lesions produced by foreign bodies in the nose is the most frequent cause of epistaxis [26]. In young people, traumatic causes of epistaxis are nasal fractures or contusions due to car or motorcycle accidents or deter aggression. Maxillofacial trauma, although rare, can produce life-threatening bleeding by lacerating the branches of the external carotid artery [27]. If nasal packing and surgical ligation of bleeding sites do not stop the bleeding, trans arterial embolization may be a treatment choice. Computer tomography with angiography can be performed to indicate the source of the bleeding, and selective embolization can be performed as distally as possible.

Delayed epistaxis sometimes appears when arterial damage leads to pseudoaneurysms. The delay period can vary from a few days to years [28], but the highest incidence is in the first three weeks from trauma [29].

### Hereditary hemorrhagic telangiectasia

Hereditary hemorrhagic telangiectasia is a disorder that produces systemic vascular dysplasia, mucosal and skin telangiectasias, affecting multiple organs. It is not a frequent pathology; the prevalence is estimated between 1 in 5000 and 1 in 8000 in Europe [30], but it is a condition that produces recurrent epistaxis and needs specific treatment techniques and technologies. Over 95% of patients with hereditary hemorrhagic telangiectasia are affected by epistaxis, the most frequent symptom. These patients will have multiple admissions to the hospital for epistaxis treatment, hemostatic drugs, and blood transfusion.

The epistaxis management depends on the severity of the bleeding. Nasal packing, monopolar or bipolar cauterization, argon plasma coagulation, and laser treatment are treatment options. To prolong the bleeding-free periods, the patient should be instructed to maintain good hygiene of the nasal cavities and avoid triggering factors [31].

The endoscopic minimally invasive approach offers several advantages over conventional techniques. It reduces morbidity and mucosal trauma, carries a lower risk of septal perforation, and can often be performed under local anesthesia, resulting in shorter hospitalization times. In patients with Rendu–Osler disease, currently available therapies can provide effective control of epistaxis, but they do not offer a definitive cure.

### Post-viral/environmental epistaxis

Spontaneous nosebleeds occurring in patients without comorbidities or anticoagulant therapy, more frequently observed in younger individuals and showing uneven distribution across the years, have led researchers to consider the role of environmental factors in triggering epistaxis. Such cases are most often reported during the winter months [32,33], influenced by low temperature, reduced humidity, and an increased predisposition to upper respiratory tract inflammation and infection. During winter, inflammatory diseases of the respiratory tract may be accompanied by nasal bleeding. Exposure to cold air and mucosal inflammation can disrupt the epithelial barrier, while nose blowing and sneezing exert additional traumatic effects on the nasal mucosa [34]. Gender differences have also been observed. The higher incidence of epistaxis in men compared with women, particularly in the 61–70 year age group, may be explained by lifestyle and behavioral factors such as smoking and alcohol consumption. In contrast, women are generally more compliant with cardiovascular therapy, which may partially account for the lower incidence in this group.

### Treatment

Most cases of epistaxis are self-limiting or can be controlled with simple maneuvers such as local compression and are often resolved at home without requiring medical care. Patients who are unable to stop the bleeding on their own seek specialized treatment. For anterior epistaxis, two main therapeutic approaches are used: (1) nasal packing with traditional dressings, inflatable packs, or balloons; and (2) cauterization when the bleeding source is identified, which may be performed chemically (silver nitrate) or with electrocautery under local anesthesia [21]. Most of the patients treated for anterior epistaxis in our clinic were responsive to nasal packing. The nasal packing was maintained for 48 to 72 hours or even several days, taking into account the severity of the bleeding and the patient's comorbidities.

Posterior epistaxis usually has an arterial source, the sphenopalatine artery or its branches, and needs advanced surgical maneuvers when nasal packing or application of hemostatic powder is ineffective. If the epistaxis is not responsive to monopolar or bipolar electrocautery, sometimes arterial ligature (internal maxillary artery, ethmoidal artery, or external carotid artery) is needed [24]. In our practice, posterior epistaxis is generally managed endoscopically with mono- or bipolar electrocautery, with bipolar cauterization preferred because it reduces the risk of septal perforation.

When no severe active bleeding is present, local vasoconstrictors can be applied and are often effective. Intravenous hemostatic agents may also be considered as an adjunctive measure. In some cases, bleeding is self-limiting, with spontaneous closure of the bleeding site observed.

## CONCLUSION

This study provides a comprehensive retrospective analysis of epistaxis cases requiring hospitalization in a tertiary ENT center, to identify major triggering factors and patterns relevant to clinical management. Based on the data collected from 260 hospitalized patients out of a total of 1,173 presenting with nosebleeds, we highlight the following key findings:

- Hypertension emerged as the most frequent associated factor, present in 63.84% of patients, supporting its well-established role in the pathophysiology of epistaxis.
- Only a quarter of all the epistaxis cases treated in the emergency room need hospitalization for advanced medical or surgical treatment.
- Patients with severe epistaxis were significantly more likely to have cardiovascular conditions. Oncological conditions are less associated with severe epistaxis.
- Anterior, less severe epistaxis was usually associated with shorter hospital stays, and nasal packing proved the most effective treatment. In contrast, severe or recurrent cases often require electrocautery as the treatment of choice.
- Postoperative bleeding was observed in 40 patients, often requiring targeted hemostatic interventions and close monitoring during recovery.
- Hereditary hemorrhagic telangiectasia (HHT) was confirmed in 11 patients, all of whom experienced recurrent episodes and multiple hospitalizations, demonstrating the chronic and complex nature of this condition.

Importantly, this study limits its analysis to clinical scenarios directly observed within our patient cohort. No assumptions or interpretations were made based on patient groups or case types not included in the study population. Future research should aim to prospectively validate these findings and explore individualized treatment protocols based on risk stratification.
